# Neuroprotection and Stroke Rehabilitation: Modulation and Enhancement of Recovery

**DOI:** 10.1155/2006/137532

**Published:** 2006-05-11

**Authors:** José Rafael Romero, Viken L. Babikian, Douglas I. Katz, Seth P. Finklestein

**Affiliations:** ^1^Department of NeurologyBoston University Medical CenterBoston UniversityBostonMAUSA; ^2^Healthsouth Braintree Rehabilitation HospitalBraintreeMAUSA; ^3^BiotrofixInc.NeedhamMAUSA; ^4^Department of NeurologyMassachusetts General HospitalBostonMAUSA

## Abstract

Recent advances in research are modifying our view of recovery after nervous system damage. New findings are changing previously held concepts and providing promising avenues for treatment of patients after stroke. This review discusses mechanisms of neuronal injury after brain ischemia and the attempts to study neuroprotection options based on such mechanisms. It also considers measures available at present to improve outcome after stroke and presents new areas of research, particularly stimulation techniques, neurogenesis and trophic factors to enhance recovery. In order to improve outcomes, medications that may be detrimental to recovery should be avoided, while symptomatic therapy of problems such as depression, pain syndromes and spasticity may contribute to better results. Continued surveillance and early treatment of complications associated with acute stroke, along with supportive care remain the mainstay of treatment for stroke patients in the recovery phase. Present research on limiting brain damage and improving recovery and plasticity enhance the prospects for better clinical treatments to improve recovery after stroke.

## 1. Introduction

Stroke is the third leading cause of mortality [13] and a leading cause of disability in the United States [81], accounting for a significant proportion of health costs every year. In 1996 the estimated lifetime cost of stroke was $40.6 billion in the US, with a large proportion spent in nursing home care [69]. Although at present few therapies are available to improve the outcome of patients with acute ischemic stroke, recent research has advanced our understanding of brain recovery, opening exciting avenues to explore clinical treatments to improve recovery.

Several factors play a role in the process of recovery after stroke including duration of the event, size and location of the infarct, presence of collateral circulation, brain plasticity, comorbidities or complications following the acute event. Functional recovery takes place to different degrees after ischemic brain injury. Not too long ago it was thought that neurons in the adult brain could not regenerate at all. This concept has now been changed by the results of recent research. Although neurons in the adult brain are not completely replaced after injury, recent studies have demonstrated the existence of populations of undifferentiated cells in the brain that may proliferate and differentiate into mature brain cells [18]. This has lead to the evaluation of several molecules that influence neuronal differentiation, such as growth factors, a promising area in the treatment of stroke.

Over the past several years our understanding of the events occurring in the brain after acute ischemia has advanced. The concept of ischemic penumbra, based on animal and human studies, has played a key role in the attempt to study further interventions to limit neuronal injury and improve outcome after stroke. The ischemic penumbra comprises the region that surrounds the core of ischemic stroke; several metabolic, molec-

ISSN 0953-4180/06/$17.00 © 2006 – IOS Press and the authors. All rights reserved ular and chemical events occur in this region that may result in further neuronal death, but offer also potential for interventions that may salvage brain cells at risk.

In the present manuscript, we briefly review the main mechanisms of neuronal injury known at present and the interventions that have been attempted to limit neuronal death. We also elaborate on new avenues for neurorehabilitation.

## 2. Mechanisms of neuronal injury

During and after an acute ischemic stroke, impaired blood supply to a region of the brain triggers a cascade of events leading to neuronal death. Among such neurotoxic events are the release of excitotoxic aminoacids such as glutamate and glycine, the production of free radicals and the induction of oxidative cellular injury, induction of apoptosis and inflammation. All these events are likely to interact and lead to cellular death. In the ischemic penumbra there is a window of opportunity to intervene and save neurons from enlarging the core of infarct. Below follows a brief review of these events.

Excitotoxicity occurs due to excessive release of excitatory aminoacids, including glutamate, aspartate and products of homocysteine and cysteine [47]. These substrates bind N-methyl-D-aspartate (NMDA) receptors, which then activate signal systems and ion channels. During ischemia, reduced cellular energy causes an increased release and decreased reuptake of glutamate. The resulting excessive stimulation of NMDA receptors produces prolonged ion channel opening and massive entry of calcium into neurons, activating other processes that lead to cell death; these secondary processes include activation of calcium dependent proteases and endonucleases, and phospholipase A2, which releases arachidonic acid from membrane lipids. Calcium accumulation in mitochondria also leads to severe damage to these organelles. Of note, even after oxygenation is reestablished calcium entry may persist due to potentiation of NMDA channel activity [53]. Finally, excitotoxicity may occur independently of glutamate, which appears mediated by cholinergic systems [62].

The drop of energy reserves (ATP, creatine phosphate) occurring during ischemia also results in ion pump dysfunction. This leads to loss of ion homeostasis, thereby causing osmotic changes that, in conjunction with failure of intracellular synthetic processes, result in cellular death.

Oxidative stress occurs as a result of imbalance between the generation of reactive oxygen species and antioxidant defense mechanisms. Oxidative stress has been documented in models of cerebral ischemia [30]. It appears to be an important factor mediating cerebral vascular changes occurring after ischemia [23]. Oxidative stress is closely related to nitric oxide (NO) pathways and the process of apoptosis. NO is a water and lipid soluble free radical synthesized by nitric oxide synthases. During ischemia, the activity of nitric oxide synthase 1 increases and is largely deleterious [42]. Oxidative stress is also an important factor regulating the signaling pathways involved in the process of apoptosis during cerebral ischemia [14].

Apoptosis is a cellular suicide program by which cells inactivate, disassemble and degrade their own structural and functional components resulting in their own death [85]. This process has been shown to occur in experimental models of ischemia: apoptotic neurons may be found more easily in the ischemic penumbra and during reperfusion [15]. The process is accompanied by activation of dormant genes and new protein synthesis. Mitochondria may transmit apoptotic signals to activate caspases, a group of proteases that regulate the process [14,85]. In mammals, these enzymes include several different proteases, which neurons can express simultaneously, in parallel and sometimes in overlapping pathways [85]; they participate in normal brain development but may also be activated pathologically by insults including ischemia. Caspase inhibitors may attenuate ischemic neuronal injury, suggesting a potential target for treatment of stroke.

Finally, inflammation is an additional important process occurring in areas of ischemia. Animal models of focal brain ischemia reveal a prominent inflammatory reaction [10]. Microglia become activated, secreting oxygen free radicals, inflammatory cytokines, complement proteins, coagulation proteins, and binding proteins [83]. Microglia may also activate the process of apoptosis. This amplifies events that cause cell death.

## 3. Trials of neuroprotection and pharmacological interventions For recovery

Based on the proposed mechanisms of neuronal injury, several trials have been carried out in an attempt to limit neuronal injury during acute brain ischemia. However, the success of these interventions to prevent further neuronal injury has been limited to date. Table 1 summarizes a list of agents that have been or are being evaluated. For the most part, these studies have not shown an improvement in neurological outcome at one to three months. The use of hypothermia as a neuroprotective measure has been promising in animal models of acute ischemic stroke [84]; human studies after cardiac arrest have also shown beneficial effects [3]. In ischemic stroke in humans, this modality is beginning to be explored, with a recent trial showing good tolerability of endovascular- induced hypothermia and a small, and non-significant, decrease in DWI (Diffusion Weighted Image) detected infarct size [21].

Several pharmacological modalities have been used to enhance recovery during the *subacute* stage after ischemic stroke. A randomized placebo controlled study of 53 patients demonstrated improved functional outcome in the group treated with 100 mg of levodopa daily, given 30 minutes prior to physiotherapy for 3 weeks, compared to physiotherapy alone [63]. Amphetamine offers the potential to improve recovery by increasing central noradrenergic activity as demonstrated in animal studies [24]; human studies of motor recovery have shown conflicting results in small randomized controlled trials. Several studies reported improvement in motor scores when amphetamine was used concomitantly with physical therapy [46,78] while others have shown no benefit when compared to placebo [66,71]. In a double-blind randomized control study assessing benefit in aphasia recovery, amphetamine was found beneficial compared to placebo [77].

It is also important to note that some medications used in the period of acute stroke may influence stroke recovery negatively. In fact, observational studies reported detrimental effects when these drugs were utilized in patients recovering from stroke [26,72]. These medications include phenobarbital, clonidine, prazosin, phenytoin, benzodiazepines and dopamine antagonists. When treating patients in the recovery phase after stroke, potential adverse effects of these medications on recovery should be considered.

In addition, treatment of associated conditions or symptoms may result in a better outcome. For instance, post-stroke depression frequently affects hemiplegic patients within the first year after a stroke and may be associated with worse recovery [50]. Fluoxetine was found to be effective in the treatment of post-stroke depression in a trial including 31 patients; however it did not have an impact on other outcome measures [80]. Citalopram has been shown to be effective in the treatment of post-stroke pathological crying, with associated improvement in depression measures [8]. Sertraline was effective preventing post-stroke depression in a study including 137 patients followed for 1 year, with benefit observed as early as 6 weeks after initiation of therapy [56]. Beneficial effects of antidepressants on functioning and motor recovery have been demonstrated, possibly independent of their effects on mood. A serotonergic antidepressant agent, trazadone, was found to improve outcome in patients with stroke based on Barthel Index scores in a double-blind trial [57]. Fluoxetine improved motor recovery after hemiplegic stroke, independent of depression measures [19]. There may be different effects of serotonergic compared to adrenergic antidepressant agents. Miyai and colleagues [48] randomized 24 patients with depression after stroke to receive either fluoxetine (serotonergic), trazadone (serotonergic) or desipramine (noradrenergic). The patients who received the serotonergic agents had significantly greater improvement in self-care and ambulation than those who received the adrenergic agent, even though all demonstrated similar improvements in depressive symptoms.

Other post-stroke problems have been successfully treated with antidepressants and other classes of medications. Central pain after stroke, which may hinder recovery, has been successfully treated with antidepressants, including amitryptiline [41] and anticonvulsants, such as lamotrigine [74]. Nimodipine, a calcium channel blocker has shown some benefit for memory dysfunction after stroke [68].

## 4. Neurorehabilitation: Enhancement and modulation of recovery

Recovery following ischemic brain injury depends on several factors. It occurs predominantly during the initial weeks to first 3 months but can continue at a slower rate during the first year [36]. Recovery during the first few weeks after stroke onset seems to be due to resolution of the initial insult associated edema, and excitotoxic and inflammatory events. Following this period, brain plasticity and reorganization seem to account for much of recovery [54,79] along with compensation, restitution, substitution of function and adaptation involving various learning processes [67].

Currently, two research fields in particular appear to be most promising in neurorehabilitation: modulation of cortical plasticity by using stimulation

Table 1
Neuroprotection trials

| Agent* | Mechanism of action | Trial | Phase/number of patients | Study features/Outcome |
|---|---|---|---|---|
| Repinotan [70] | Serotonin 5HT1A receptor agonist, interferes with ischemia induced cell death | BRAINS | II $N = 240$ | Within 6hours; safe; no significant benefit |
| Nimodipine [33] | Calcium channel antagonist | VENUS | $N = 454$ | Within 6hours; terminated early due to lack of benefit |
| Dextrorphan [6] | NMDA antagonist | | $N = 66$ | Within 48 hours; Side effects limiting use |
| Selfotel [20] | NMDA antagonist | ASSIST | $N = 476$ | Increased mortality, terminated early |
| Aptiganel [7] | NMDA antagonist | | II/III $N = 628$ | Within 6 hours. Terminated early due to benefit/risk concerns |
| Gavestinel (GV150526) [61] | Indirect NMDA antagonist (Glycine antagonist) | GAIN | $N = 1367$ | Within 6hours, Yes t-PA. No improvement but tolerated well |
| Magnesium sulfate [52] | Increase regional blood flow, NMDA antagonist, Ca channel antagonist | IVMgET IMAGES | $N = 2589$ | No benefit within 21 hours No benefit within 12 hours |
| Nalmefene (cervene) [16] | Narcotic R antagonist, decreases levels of excitatory aminoacids | | III $N = 368$ | No efficacy IV within 6 hours |
| Lubeluzole [22,28] | Sodium channel blocker, decreases levels of NO | | $N = 721$ | Within 6 hours suggestive of benefit. |
| | | | $N = 1786$ | Within 8hours, no efficacy |
| Sipatrigine [51] | Sodium channel blocker | | $N = 27$ | Within 12 hours; significant neuropsychiatric effects |
| Clomethiazole [43, 76] | GABA agonist | CLASS I | $N = 1198$ | No efficacy within 12 hours; in subgroup improved 37% in large stroke patients |
| Nitrones [40] | Free radical scavenger | | $N = 150$ | More frequent ICH and death |
| ENLIMONAB [5] | Monoclonal antibody to ICAM-1, Block intercellular adhesion molecules | | $N = 625$ | Higher mortality (within 6hours for 5 days) |
| Abcximab [1] | Platelet aggregation inhibitor | | $N = 74$ | Within 24 hours, possible benefit but slightly more frequent ICH |
| Citicoline [17] | Membrane stabilization, decreases free radical formation | | III $N = 899$ | No efficacy but posthoc analysis showed improved outcome in severe strokes |
| Neutrophil inhibitory factor UK 279,276 [37,39] | | ASTIN | $N = 966$ | Within 6 hours, terminated early for futility |
| Fiblast (Trafermin) [11] | Basic fibroblast growth factor | | $N = 286$ | Within 6 hours. US trials terminated due to side effects, European trial (lower doses) suggested possible benefit for stroke recovery |
| Magnesium sulfate [38] | NMDA receptor antagonism | FAST-MAG | III $N = 1298$ | $<$2hrs No t-PA. Ongoing |
| ONO-2506 [4] | Astrocyte modulation | RREACT | IIb/III $N = 1320$ | $<$6hrs Yes t-PA |
| Glyceryl trinitrate (a nitric oxide donor) [2] | Lowering blood pressure, improving cerebral perfusion, and neuroprotection. | ENOS | $N = 5000$ | Ongoing |

*References in parenthesis. ICH = Intracerebral hemorrhage.

techniques and restoration of cellular populations. Non-pharmacological interventions that may improve outcome include repetitive arm stimulation [25], transcranial magnetic stimulation (TMS) [44], and physical and occupational therapy. Prior studies have shown that changes in environment may lead to structural changes in the brain. These include increase in brain weight, cortical thickness, neuronal size, dendritic complexity, synaptogenesis and the generation of new neurons, glia, and blood vessels [27]. Return of function after stroke may occur spontaneously, but some interventions have improved outcome. In particular, functional recovery in stroke can be enhanced by environmental manipulation such as constraint limb induced movement therapy [29]. This technique utilized restraint of the unaffected arm and repetitive practice with the affected arm. It is yet to be determined how much of the enhancement of function is due to the mass practice of the paretic arm versus inhibition of use of the non affected side.

TMS has been utilized as a technique to study brain reorganization after stroke, as well as a prognostic tool to assess recovery following stroke [31]. In addition, TMS in normal volunteers has been used to modulate cortical excitability. Lasting intracortical excitability changes have been shown in normal volunteers after TMS [60]. In preliminary studies these changes have been reflected in lasting effects in cognitive function; these include improved spatial attention to ipsilateral targets [32], improved analogic reasoning [12] and reaction time [55]. A recent study in patients with stable chronic stroke using central and peripheral stimulation resulted in an improvement in functional and neurophysiological measures [73]. The presumed underlying mechanism was brain plasticity likely involving enhanced synaptic efficacy and decreased local cortical inhibition. Although the studies mentioned above have been limited, further research might result in protocols that could modulate cortical reorganization in a predictable manner. TMS offers the potential to suppress maladaptive reorganization (i.e. post-stroke dystonia) or enhance beneficial plasticity [65] in stroke rehabilitation.

Another field that has shown recent progress involves the production of new neurons (neurogenesis). This may be achieved by using endogenous precursor cells or stem cell transplantation. Neurogenesis has been shown to occur in animals as well as in adult humans [49]. There is potential for regeneration in the adult mammalian brain, although to a lesser degree than in the neonate. Progenitor cells with potential to differentiate into neurons have been found in the temporal lobe's subventricular zone in adult humans [34]. In experimental animal studies of stroke, increased progenitor cell proliferation occurs in the subventricular zone and the hippocampal dentate gyrus [75]. Some of these cells are immature neurons and may migrate to the olfactory bulb following their natural migration route, but also to the periphery of the infarct [75]. Newly formed neurons in the hippocampus remain in site. These studies show enormous potential for interventions improving neurologic recovery after stroke. However the cellular environment in the adult human brain may not be optimal for regeneration; factors such as absence of neurotrophic factors and presence of inhibitors in oligondendrocytes may account for this [35].

Recent studies have explored the role of neurotrophic factors in promoting cellular regeneration [35,59]. Trophic factors are important in differentiation and survival of neural elements. These molecules interact with specific receptors and initiate a variety of cellular signaling systems. The probable mechanisms by which growth factors may enhance recovery include stimulation of new sprouting and synapse formation in undamaged parts of brain, and stimulation of progenitor cells. Animal models of stroke have demonstrated improved recovery of function following treatment with basic fibroblast growth factor (bFGF) [59]. This benefit may have been mediated by different effects of bFGF, including enhancement in axonal sprouting in the periphery of the infarct, and stimulation of proliferation, migration, and differentiation of progenitor cells [59]. Osteogenic protein 1 is another growth factor that has shown improved sensorimotor recovery in rats when given during the first five days after a stroke [58]. Vascular endothelial growth factor studies in animals have shown conflicting results [59]. Granulocyte colony stimulating factor (GCSF) resulted in increased number of hematopoietic cells in the ischemic hemisphere, with some seemingly differentiating into neurons in a prior study [82]. GCSF also resulted in improved function and reduced infarct volume in a study in rats [64].

Some factors, such as ciliary neurotrophic factor (CNTF) and brain-derived neurotrophic factor (BNDF), influence survival of motor neurons. Clinical trials in humans are beginning to be evaluated. Ongoing trials are exploring the effects of CNTF. However, trials addressing the use of neurotrophic factors in stroke recovery have not been undertaken yet.

Transplantation of cells to restore function is a promising nascent field. Most of the new neurons generated in animal stroke models die during the first few weeks, with only about 0.2% of the initial neurons being replaced [9]. Thus, exogenous cells may provide an additional substrate to replace neurons, with the theoretical potential to result in recovery of function. At present many basic issues remain unsolved but this is a rapidly growing field that needs careful exploration as an option for stroke recovery in humans.

## 5. Where do we stand in our daily practice?

Translation of research findings into practice takes time and requires support from several studies. At present, in neurorehabilitation services particular attention toward the surveillance, assessment and treatment of stroke comorbidities, vascular risk factors, and associated complications is recommended. Supportive care remains the mainstay of rehabilitative stroke care. While physical, occupational and speech therapy have proven benefit in stroke recovery, pharmacological interventions are increasingly becoming a part of daily practice. In the rehabilitation setting, pharmacologic agents are most commonly used to treat stroke-associated symptoms and problems, particularly the use of antidepressants (e.g. SSRIs) for the treatment of depression, anticonvulsants (e.g. gabapentin, lamotrigine) and tricyclic antidepressants (e.g. desipramine and amitriptyline) to treat pain syndromes, medications for spasticity (e.g. botulinum toxin injections, baclofen, tizanidine) and anticholinergic medications for bladder spasticity (e.g. tolterodine and oxybutynin). Despite possible benefit of amphetamines and dopaminergic agents in accelerating recovery of motor and language impairments after stroke in some human studies, stimulant medications have been slow in entering routine stroke rehabilitation practice. A recent Cochrane review concluded that the present level of evidence does not support routine use of stimulants for stroke recovery [45]. The use of these medications remains an option for patients with stroke but larger, randomized, and controlled trials are necessary to satisfy remaining questions about the effects of stimulant medications on stroke outcome before widespread use is recommended for routine use. Use of stimulant and dopaminergic medications (e.g. methylphenidate, amphetamines, amantadine, bromocriptine) also remains an option for patients with persistent deficits in arousal and attention after stroke, for instance infarcts and hemorrhages damaging mesencephalic, diencephalic and mesial frontal areas. Physicians and other health care providers must encourage the participation of patients in research trials in order to advance knowledge about stroke recovery and find options that are more meaningful for the quality of life of stroke patients.

In conclusion, neuroprotection and neurorehabilitation are exciting growing fields. Neuroprotection may in the future have an important role in improving recovery in the acute phase after stroke. In the subacute and later phases of recovery, other approaches such as modulation of neurogenesis with growth factors, implantation of exogenous precursor or stem cells, or techniques such as TMS to modulate brain plasticity, might result in improved recovery. It is likely that a combination of measures will be utilized to improve outcome following stroke.
